# Multifunctional Ag-Poly(*N*-isopropylacrylamide/itaconic Acid) Hydrogel Nanocomposites Prepared by Gamma Irradiation for Potential Application as Topical Treatment Dressings

**DOI:** 10.3390/polym16223211

**Published:** 2024-11-19

**Authors:** Jelena Spasojević, Milica Milošević, Sašenka Vidičević-Novaković, Jelena Tasić, Petar Milovanović, Marija Djurić, Dragan Ranković, Zorica Kačarević-Popović, Aleksandra Radosavljević

**Affiliations:** 1 Vinča Institute of Nuclear Sciences, National Institute of the Republic of Serbia, University of Belgrade, Mike Petrovića Alasa 12-14, Vinča, 11351 Belgrade, Serbia; jelenas@vin.bg.ac.rs (J.S.); milicam@vin.bg.ac.rs (M.M.); dragan.rankovic@vin.bg.ac.rs (D.R.); zokkacpop@gmail.com (Z.K.-P.); 2 Institute of Medical and Clinical Biochemistry, Faculty of Medicine, University of Belgrade, Pasterova 2, 11000 Belgrade, Serbia; sasenka.vidicevic@med.bg.ac.rs (S.V.-N.); jelena.tasic@med.bg.ac.rs (J.T.); 3 Center of Bone Biology, Institute of Anatomy, Faculty of Medicine, University of Belgrade, Dr Subotića 4/2, 11000 Belgrade, Serbia; drpmilovanovic@gmail.com (P.M.); marijadjuric5@gmail.com (M.D.)

**Keywords:** γ-irradiation, hydrogel nanocomposites, Ag nanoparticles, microstructural properties, Ag^+^ ion release, antibacterial properties, cytotoxicity

## Abstract

Today, hydrogel dressings that can protect injury sites and effectively promote healing have become highly desirable in wound management. Therefore, multifunctional silver-poli(*N*-isopropylacrylamide/itaconic acid) (Ag-P(NiPAAm/IA)) hydrogel nanocomposites were developed for potential application as topical treatment dressings. The radiolytic method, used for the crosslinking of the polymer matrix as well as for the in situ incorporation of silver nanoparticles (AgNPs) into the polymer matrix, enables the preparation of hydrogel nanocomposites without introducing harmful and toxic agents. Moreover, materials produced using γ-irradiation are simultaneously sterilized, thus fulfilling one of the basic requirements regarding their potential biomedical applications. The NiPAAm/IA ratio and the presence of AgNPs influenced the microstructural parameters of the investigated systems. Increasing the IA content leads to the formation of a more porous polymer matrix with larger pores, while the incorporated AgNPs act as additional junction points, decreasing the porosity and pore size of the resulting nanocomposite hydrogels. Swelling studies showed that most investigated systems uptake the fluids from their surroundings by non-Fick diffusion. Further, the Ag^+^ ion release, antibacterial activity, and cytotoxicity of Ag-P(NiPAAm/IA) hydrogel nanocomposites were examined to evaluate their biomedical potential. All hydrogel nanocomposites showed an initial burst release of Ag^+^ ions (useful in preventing bacteria adherence and biofilm formation), followed by a slower release of the same (ensuring sterility for longer use). An antibacterial activity test against *Escherichia coli* and *Staphylococcus aureus* showed that Ag-P(NiPAAm/IA) hydrogel nanocomposites, with silver concentrations around 10 ± 1 ppm, successfully prevent bacterial growth. Finally, it was shown that the investigated hydrogel nanocomposites do not exhibit a cytotoxic effect on human keratinocyte HaCaT cells. Therefore, these multifunctional hydrogel nanocomposites may promote wound repair and show promising potential for application as functional wound dressing.

## 1. Introduction

In recent years, the technological need for new materials has led to creative approaches to the synthesis and generation of structures, properties, and functions in the field of nanocomposite systems based on polymeric hydrogels and metal nanoparticles. Considering their unique properties and synergistic effects, these materials have become a very attractive class of new materials in various fields of science and technology.

Nowadays, great emphasis has been placed on the development of suitable wound dressings with appropriate antibacterial effects for topical skin treatment, which could be able to protect the injury site from further insult, contamination, and infection, but also to provide gas and nutrient permeability, exudate absorption, and facilitate cells’ adhesion, growth, and migration to achieve the re-epithelialization of the skin. From this point of view, hydrogels can probably be considered as one of the best choices for this purpose, especially when they contain active substances such as antibacterial agents and/or drugs [1,2,3,4,5]. Due to their unique crosslinked structure, porous morphology, ability to absorb a large amount of water or biological fluid, and similarity to living tissues, hydrogels behave as containers of drugs which control the release kinetics of active substances to the surrounding environment. Generally, the controlled release of a drug from hydrogel may be regarded as a three-step process. In the beginning, hydration occurs through the absorption of dissolution media into the hydrogel, which is followed by polymer chains’ relaxation or erosion. The last step is the diffusion of dissolved drugs either through the eroded parts or from the hydrated polymer matrix to the surrounding medium [6].

Among the numerous nanoparticles that have been used in nanocomposite systems, the most promising have become silver nanoparticles (AgNPs), particularly in the field of potential biomedical application, due to their ability to prevent the growth and development of single-celled organisms meaning that they can be used as a therapeutic agent against bacteria, fungi, or viruses [1]. Furthermore, AgNPs also possess strong antiseptic and anti-inflammatory properties, as well as a low systemic cytotoxicity effect. It is well known that AgNPs with a radius in the range of 7–20 nm show excellent antibacterial activity by inhibiting cell proliferation and replication [7,8]. Today’s market has plenty of AgNP-containing industrial and medical products, including textiles, food packing, cosmetics, water disinfectants, implant coatings, wound dressings, etc. It was reported that AgNPs are ingredients in around 30% of commercially available nano-based products, indicating their broad usability and safety in human applications [9]. However, AgNPs may be harmful, and it is essential to properly assess the potential risks associated with their administration, such as toxicity safety issues, this being the most important one. Numerous studies have shown a narrow gap between their bactericidal efficacy and their toxicity to mammalian cells.

The skin is the largest human organ and plays a vital function in defense against injury and microbial invasion. Wound healing is a dynamic, complex, and multistep process that is usually divided into four successive and overlapping phases (hemostasis, inflammation, proliferation, and remodeling) involving the simultaneous activity of different cell types, biological mediators, and tissues. During wound healing, the re-epithelialization is an important process which starts with the migration of keratinocytes from the wound edge to generate a thin layer of cells over the wound. Along with the migration, the proliferation of the keratinocytes also takes place to ensure the efficient restoration of skin integrity [3,10,11,12]. Since the outer epidermis of the skin consists of 90% keratinocytes, these cell types are the most exposed to the impact of AgNPs, so this influence should be assessed more extensively [13,14]. Therefore, the implementation of AgNP-based wound dressing is faced with many challenges, and the development and optimization of the nanocomposites that will deliver the antibacterial silver within the therapeutic non-toxic dose range (0.78–12.5 ppm) can significantly increase the efficiency of their application [15]. In addition, intelligent hydrogels have received considerable interest due to their excellent responsiveness to small changes in their surrounding environment such as changes in temperature, pH, oxygen and glucose levels, etc. As smart dressings, they can respond to one or more stimuli and thus actively contribute to the wound healing process. Probably the most investigated are thermo- and pH-sensitive hydrogels because the temperature and pH value are the two parameters that are most frequently changed in physiological systems. In the case of wounds, the local change in the temperature and pH values arises as the organism responds to infliction, inflammation, and infection [10,11,16]. Poly(*N*-isopropylacrylamide) (PNiPAAm) is the most studied temperature-sensitive polymer, with a thermosensitivity in the range of physiologically relevant temperatures (around 32 °C), which can be tailored by copolymerization [17]. In wound management, thermosensitive hydrogels can be used as carriers for antimicrobial agents, different drugs, and biologically active agents such as growing factors, cells, or proteins that can be locally delivered to activate immune cells, and promote and accelerate wound healing [10]. Furthermore, numerous research activities are directed towards the development of mechanoactive wound dressings which, due to their thermosensitive shrinkage and enhanced adhesivity, exert contractile forces strong enough to induce wound closure [18].

Silver-based hydrogel nanocomposites can be produced by various methods, but the growing emphasis on environmental protection has led to the development and improvement of an eco-friendly approach to the synthesis of AgNP-hydrogel nanocomposites. Bearing in mind that the principles of the radiation-chemical method of synthesis (using gamma irradiation) are based on biologically harmless and biocompatible radiolytic products of water, this method combines the advantages of environmental reagents with the simultaneous stabilization of particles in the polymer matrix. In addition, it is a fast and easily controlled method of synthesis, but one of the most important benefits is the possibility of the synthesis and sterilization of products in one technological step, which is extremely important for biomedical applications [19,20,21,22,23].

Our previous work deals with radiolytically fabricated Ag-P(NiPAAm/IA) hydrogel nanocomposites and their physicochemical characterization, and the preliminary results for the test sample have demonstrated favorable antibacterial activity [17]. This study aims to go a step forward and evaluate properties of these hydrogel nanocomposites that are relevant to their potential use as topical treatment dressings in wound healing. After gamma irradiation-induced synthesis, the produced Ag-P(NiPAAm/IA) hydrogel nanocomposites were sterilized and ready for biomedical application as topical treatment dressings, which represents one of the innovations of this research. Moreover, silver release, antibacterial activity, and cytotoxicity tests were performed, and the obtained results provide deeper insight into the biomedical potential of the investigated hydrogel nanocomposites. The Ag^+^ ion release mechanism was determined by fitting experimentally obtained data with several commonly used kinetics models of drug release (first-order, Higuchi, Korsmeyer–Peppas, Kopcha, and Peppas–Sahlin models). It is well known that AgNPs possess excellent bactericidal effects against different strains of bacteria, but they can also be toxic to mammalian cells. Therefore, the antibacterial potential of AgNPs against Gram-negative (*Escherichia coli—E. coli*) and Gram-positive (*Staphylococcus aureus—S. aureus*) bacteria as well as their toxicity on the human keratinocytes HaCaT cell line were evaluated. All in all, this work shows that the investigated Ag-P(NiPAAm/IA) hydrogel nanocomposites have huge potential to be used as multifunctional dressings for local therapy, combining the functions of continuous protection and treatment with biocompatibility, but also thermo- and pH sensitivity originating from their polymer matrix.

## 2. Materials and Methods

### 2.1. Materials

N-isopropylacrylamide (NiPAAm) was obtained from Sigma-Aldrich (Saint Louis, MO, USA), while itaconic acid (IA) was a product of Across Organic (Antwerpen, Belgium). Silver nitrate (AgNO_3_) and 2-propanol (C_3_H_8_O), which are used for AgNPs’ preparation, were purchased from Merck (Rahway, NJ, USA). Simulated body fluid (SBF), with ion concentration approximately equal to that in human blood plasma, was prepared according to the standard procedure [24]. Potassium dihydrogen phosphate (KH_2_PO_4_) and potassium hydrogen phosphate (K_2_HPO_4_) were obtained from Kemika (Zagreb, Croatia). All chemicals were used without purification (commercial product with the highest available purity), except NiPAAm, which was recrystallized from benzene/n-hexane mixture (35/75) [25]. Solutions were prepared with ultra-pure distilled water obtained from Milli-Q water system (Millipore Corporation, Wicklow, Ireland) and were saturated with argon gas (purity of 99.5%, Messer Tehnogas, Belgrade, Serbia) to remove oxygen. All systems were exposed to gamma irradiation (^60^Co source) in closed cells under ambient conditions.

### 2.2. Synthesis of Ag-P(NiPAAm/IA) Hydrogel Nanocomposites

Series of P(NiPAAm/IA) hydrogels were prepared by varying the ratios of the monomers. Different amounts of IA were added into NiPAAm solutions (10 wt%) to obtain mixtures of NiPAAm/IA with the weight ratios 100.0/0.0, 98.5/1.5, 97.0/3.0, and 95.5/4.5. The solution mixtures were bubbled with argon for 30 min, poured into specially designed glass molds, and exposed to gamma irradiation up to an absorbed dose of 50 kGy (dose rate 0.5 kGy/h). After the irradiation process was completed, hydrogels were cut into discs and immersed in distilled water (changed daily for one week) in order to remove all unreacted residues.

The obtained P(NiPAAm/IA) hydrogel samples (discs with diameter of 10 mm and thickness of 4 mm) were left to swell in 40 mL of AgNO_3_ (1.0 × 10^−2^ mol/dm^3^) and 2-propanol (0.2 mol/dm^3^) solution previously saturated with argon. The swelling process was carried out at room temperature, in the dark (to avoid photosensitivity of AgNO_3_), for 48 h. These samples were exposed to gamma irradiation up to an absorbed dose of 18 kGy (dose rate 17 kGy/h) to perform a reduction of Ag^+^ ions and formation of AgNPs within the hydrogels.

### 2.3. Methods of Characterization

#### 2.3.1. Scanning Electron Microscopy (SEM)

The internal morphology of hydrogel nanocomposites as well as the morphology of incorporated AgNPs was investigated by scanning electron microscopy (SEM). Prior to SEM observation, equilibrium swelled nanocomposite hydrogels were frozen for 48 h at −20 °C, then lyophilized in Martin Christ Alpha 1–2 LDplus freeze-dryer (Osterode am Harz, Germany) for 48 h at temperature of −32 °C and pressure of 0.310 mbar, and then fractured. Cross-sections of hydrogel samples were analyzed by field-emission scanning electron microscopy (FE-SEM, Tescan MIRA 3XMU, Brno, Czech Republic), operated at an accelerating voltage of 8 kV, while the shape and size of AgNPs were analyzed using JEOL JSM 6610LV SEM (Tokyo, Japan), operated at an accelerating voltage of 20 kV. Prior to observation, fractured samples were coated with a thin gold layer (around 15 nm) in LEICA SCD005 nebulizer (Wetzlar, Deutschland).

#### 2.3.2. Micro-Computed Tomography (Micro-CT)

Internal morphology of samples was investigated by using micro-computed tomography (micro-CT), followed by three-dimensional reconstruction. Freeze-dried samples were scanned by a high-resolution micro-CT system (Skyscan 1172, Bruker, Kontich, Belgium) with an isotropic resolution of 2.45 μm. The scanning parameters were set at 60 kV and 167 μA, exposure time of 480 μs, 2K camera binning, 0.25° rotation step, and frame averaging of 2. The reconstruction of the projection images was accomplished using NRecon software (version 1.6.9.8) with the InstaRecon engine (version 2.0.2.5), with appropriate corrections for thermal drift, misalignment, ring artifact, and beam hardening. Ct. An program version 1.14 (Skyscan, Kontich, Belgium) was used for the quantitative analysis of the data. The microstructural parameters of investigated hydrogels were evaluated using automatic 3D analysis.

#### 2.3.3. X-Ray Diffraction (XRD)

Microstructural properties of Ag-P(NiPAAm/IA) xerogel nanocomposites (samples drayed at the ambient condition) were investigated by X-ray diffraction (XRD) measurements performed on Bruker D8 Advance Diffractometer (Kontich, Belgium) (CuKα1 radiation, λ = 0.1541 nm). The diffractograms were recorded in 2θ range from 10° to 90°, with exposure of 10 s and step of 0.05°.

#### 2.3.4. Swelling Studies

Swelling measurements were performed in distilled water and SBF (simulated body fluid) at 37 °C. The xerogel discs (diameter ≈ 4 mm, thickness ≈ 1.5 mm) were immersed in a swelling medium, and swelling capacity was monitored gravimetrically at predetermined time intervals to determine swelling degree (*SD*) and equilibrium swelling degree (*SD_eq_*) using following equations:

(1) $$SD=\left(W_{t}-W_{0}\right)/W_{0}$$

(2) $${SD}_{eq}=\left(W_{eq}-W_{0}\right)/W_{0}$$

where *W_t_* is weight of swollen hydrogel at predetermined time intervals, *W_eq_* is weight of swollen hydrogel at equilibrium, and *W_0_* is the initial weight of the xerogel [17]. All swelling measurements were performed in triplicate and average values were presented.

#### 2.3.5. *In Vitro* Silver Release Study

The release of Ag^+^ ions from the Ag-P(NiPAAm/IA) hydrogel nanocomposites was examined at 37 °C in phosphate (KH_2_PO_4_/K_2_HPO_4_) buffer solution (PBS, pH = 7.4). Nanocomposite disc samples (diameter ≈ 8 mm, thickness ≈ 4 mm) were immersed in 10 mL of PBS, which was switched at predetermined time intervals with a new equal volume of PBS to maintain the perfect sink condition. Furthermore, the total content of silver within the Ag-P(NiPAAm/IA) hydrogel nanocomposites was determined upon treatment in HNO_3_ (1:1 v/v) to induce the oxidation of all AgNPs into Ag^+^ ions. The Ag^+^ ion concentration was determined by argon-stabilized arc emission spectrometry [26,27] by measuring the intensity of the spectral emission lines of Ag I 328.068 nm, followed by applying analytical curve method. All Ag^+^ release experiments were performed in triplicate and average values were considered.

#### 2.3.6. Antibacterial Properties

*Escherichia coli* and *Staphylococcus aureus* were selected for antibacterial tests of Ag-containing hydrogel nanocomposites as representative strains of Gram-negative and Gram-positive bacteria, respectively. The *in vitro* antibacterial activity was investigated by the disk diffusion and optical density methods.

For the disk diffusion method, Petri dishes covered with solidified nutrition agar base (2% agar) were inoculated with *E. coli* (ATTC 25922) and *S. aureus* (ATTC 25923) inside the layer of the top agar (0.7% agar). Each bacterial culture (20 mL of 18 h-old bacteria) was added to 15 mL of the top agar solution, put onto the nutrition agar base, and left to solidify. Hydrogel discs (diameter ≈ 8 mm, thickness ≈ 4 mm) of P(NiPAAm/IA) and Ag-P(NiPAAm/IA) were put in the made wells then incubated at 37 °C for 24 h, and the appearance of a clear zone around the disc specimens was monitored.

In the optical density method, *E. coli* and *S. aureus* were seeded in 24-well plates, each in 1 mL of RPMI-1640 medium consisting of 5% FBS (fetal bovine serum), 1% glutamate, 1% sodium pyruvate, and 4.5 g/L glucose. Three different concentrations of both bacterial cultures (2 × 10^4^, 1 × 10^5^, and 5 × 10^5^ CFU/mL) were seeded and treated with P(NiPAAm/IA) hydrogels and Ag-P(NiPAAm/IA) hydrogel nanocomposites. The untreated bacterial cultures were used as control samples. After 24 h of incubation at 37 °C, the turbidity in each well was determined by TECAN Sunrise reader at 405 nm (OD405). The percentage of surviving bacteria was obtained by comparing OD405 values for treated and nontreated bacteria. All experiments were performed in triplicate and mean values were presented.

#### 2.3.7. Cytotoxicity Testing

To evaluate the biocompatibility of the investigated systems, the *in vitro* cytotoxicity of AgNPs was tested on human keratinocytes HaCaT cell line. After defrosting, HaCaT cells were sub-cultivated in an incubator at 37 °C for 7 days, in a humidified atmosphere with 5% CO_2_. They were cultivated in DMEM (Dulbecco’s Modified Eagle Medium) supplemented with 20 mM HEPES medium (4-(2-hydroxyethyl)-1-piperazineethanesulfonic acid), 10% FBS, 4 g/L glucose, 2 mM L-glutamine, 5000 U/mL penicillin, and 5 mg/mL streptomycin. Then, HaCaT cell lines were seeded in a 24-well plate (25 × 10^4^ cells/well) and, after preparation, were treated with P(NiPAAm/IA) hydrogels and Ag-P(NiPAAm/IA) hydrogel nanocomposites with a silver concentration of around 10 ± 1 ppm. The untreated HaCaT cell line was used as a control. All incubation processes were performed at 37 °C for 24 h. The viability of cultivated HaCaT cells was determined by the well-defined protocol for crystal ciolet assay, measuring the optical density of each well at 570 nm (OD570) with a TECAN Sunrise plate reader. The viability of HaCaT cells was determined by comparing the OD570 values of treated cells with the OD570 values of the nontreated cells [28]. All OD570 measurements of treated cells were done in triplicate and average values were considered.

## 3. Results and Discussion

A series of P(NiPAAm/IA) hydrogels and Ag-P(NiPAAm/IA) hydrogel nanocomposites were synthesized by gamma irradiation-induced copolymerization, crosslinking, and the reduction of metal ions, according to the previously reported procedure. Briefly, in the first step, the solutions of NiPAAm/IA (with the weight ratios 100.0/0.0, 98.5/1.5, 97.0/3.0, and 95.5/4.5) were subjected to gamma irradiation to simultaneously induce copolymerization and crosslinking, and hydrogels with gel contents of 97.3%, 96.8%, 95.3%, and 94.9%, respectively, were produced. Then, in the second step, the obtained P(NiPAAm/IA) hydrogels were swelled with Ag^+^ ions and exposed to gamma irradiation to perform the reduction of ions and formation of AgNPs within the crosslinked matrices. As a result, transparent yellow-colored Ag-P(NiPAAm/IA) hydrogel nanocomposites were obtained, with silver contents of 0.029 wt% in 100.0/0.0 and 98.5/1.5, 0.027 wt% in 97.0/3.0, and 0.026 wt% in 95.5/4.5 polymer matrices [17].

### 3.1. Microstructural Characterization of Crosslinked Systems

It is well known that one of the main characteristics of hydrogels is their highly porous structure, capable of absorbing and retaining a large amount of fluid and active substance between the crosslinked polymer chains. Therefore, the comprehensive morphological characterization of these porous materials is essential. In order to examine the internal morphologies of the investigated systems, a combination of two different techniques was applied: FE-SEM and micro-CT analysis.

**Figure 1:**
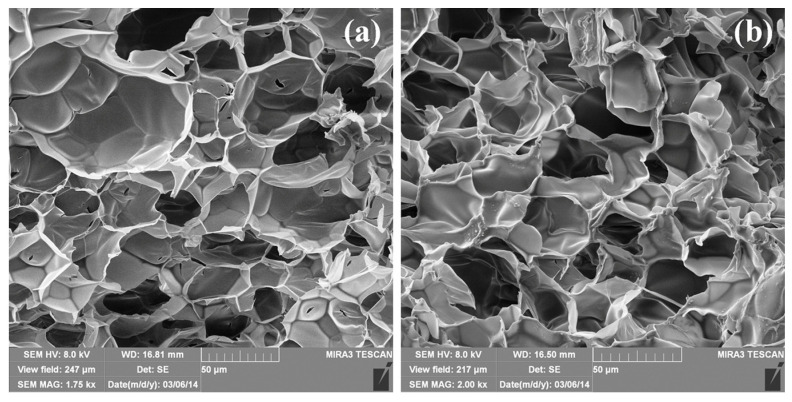
FE-SEM micrographs of (**a**) P(NiPAAm/IA) 95.5/4.5 and (**b**) Ag-P(NiPAAm/IA) 95.5/4.5.

From FE-SEM micrographs (Figure 1), the highly porous structure of the investigated samples, with different sizes of micropores, is obvious. The addition of IA as a comonomer leads to an increase in pore size in comparison with the PNiPAAm homopolymer hydrogel, but some sort of honeycombed morphology is preserved [17]. On the other hand, the incorporation of AgNPs into the polymer network (Figure 1b) has no significant influence on the internal morphology or pore size compared with the neat polymer matrix (Figure 1a), which can be very important for the possible application of such materials.

**Figure 2:**
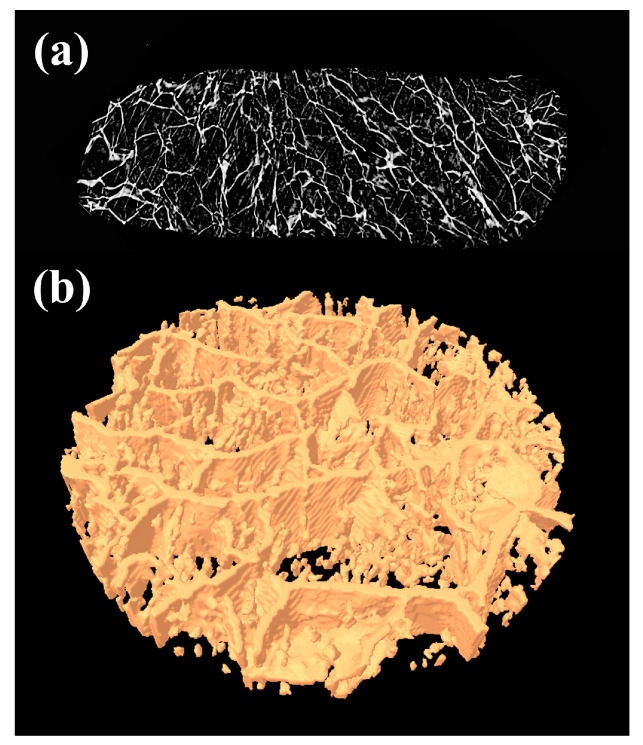
Representative micro-CT images of P(NiPAAm/IA) 100.0/0.0 hydrogel: (**a**) 2D cross-section and (**b**) 3D reconstruction.

The pores with smooth and non-porous walls, as well as their connectivity, are clearly visible on the FE-SEM micrographs, but this technique does not provide a wider quantitative evaluation regarding their microstructural parameters. To overcome these shortcomings, micro-CT, as a non-destructive technique, was used to obtain quantitative insights regarding the overall internal microarchitecture of the porous materials without causing any alteration to the sample. The principles of micro-CT–based material characterization involve obtaining a number of 2D cross-section images (Figure 2a) and the subsequent 3D reconstruction of the samples (Figure 2b), followed by 2D and 3D morphometric analysis via fitting algorithms [29] to obtain different microstructural parameters. The advantage of micro-CT analysis is that the combination of data from the 2D segmentation and 3D reconstruction of samples enables the calculation of microstructural parameters regarding the entire volume of the investigated samples. Due to the resolution limitations of the method and measuring equipment, the pore size obtained through this analysis should be used for different systems comparisons, and not treated as an absolute value [30].

The porosity and the pore sizes, as well as their distribution, are the main properties of hydrogel systems. The microstructural parameters of P(NiPAAm/IA) hydrogels and Ag-P(NiPAAm/IA) hydrogel nanocomposites are presented in Table 1. The highly porous structure of the investigated systems, revealed by FE-SEM, was confirmed, and the obtained data show that the porosity increases by around 5.5%, 11.4%, and 14.5% for copolymer matrices with 1.5 wt%, 3.0 wt%, and 4.5 wt% IA content, respectively, in comparison with PNiPAAm homopolymer matrix. As expected, increasing the porosity of polymer matrices leads to an increase in their mean pore size and a decrease in their mean wall thickness, causing a decrease in the polymer volume fraction (the fraction of polymer matrix in the total volume of the examined sample [31]). On the other hand, comparing the Ag-hydrogel nanocomposites with the corresponding neat polymer matrices, it can be noticed that the incorporation of AgNPs into the system decreases the values of porosity, mean pore size, and mean wall thickness, and increases the value of the polymer volume fraction (Table 1). Further, the connectivity density (the number of connected walls between the pores per unit volume of the sample [32]) decreases with an increasing IA content in the polymer network, and the values are higher for the hydrogel nanocomposites (Table 1), which is expected considering the values of the porosity and polymer volume fraction. These data indicate that some changes in the P(NiPAAm/IA) hydrogels’ original structure occur during the incorporation of AgNPs into the polymer matrix.

**Figure 3:**
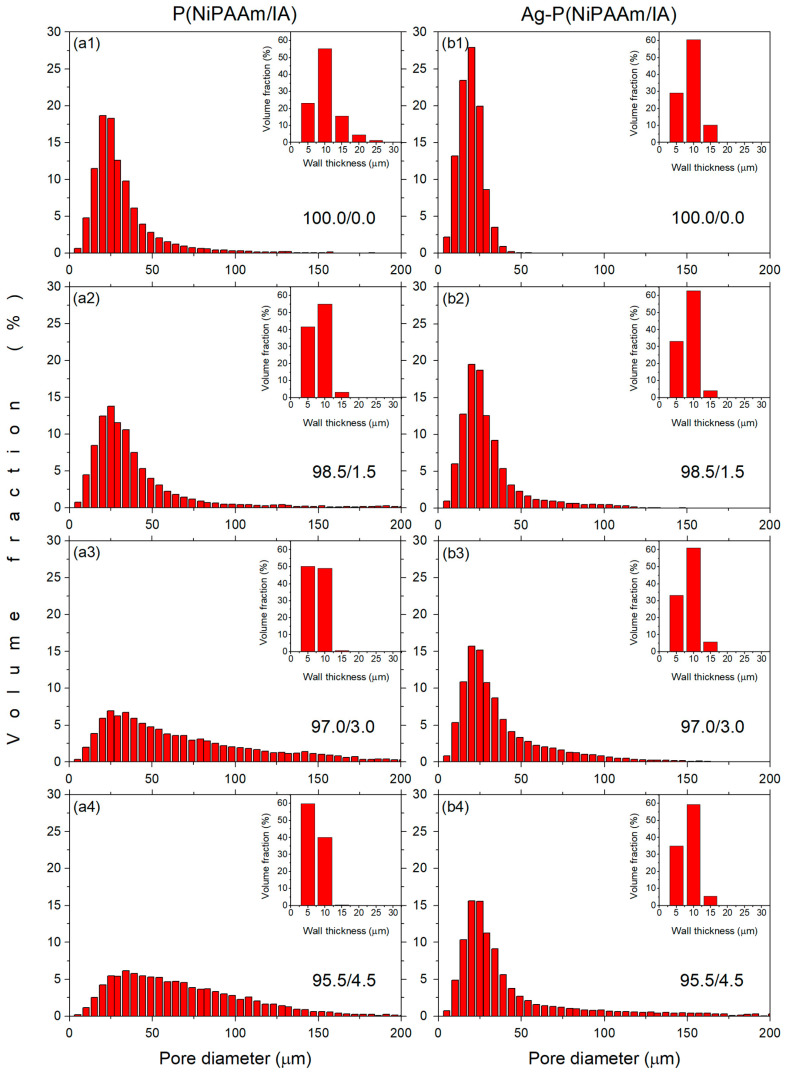
Pore size distribution and wall thickness distribution (insets) for P(NiPAAm/IA) hydrogels (**a1**–**a4**) and Ag-P(NiPAAm/IA) hydrogel nanocomposites (**b1**–**b4**) obtained by micro-CT analysis.

A more comprehensive study of porosity regarding the pore size distribution and wall thickness distribution is presented in Figure 3. It is clearly visible that widening of the pore size distribution for P(NiPAAm/IA) hydrogels occurs with increases in the IA content (Figure 3(a1–a4)). The volume fraction of smaller pores (less than 50 μm) decreases, leading to an increase in the mean pore size and a higher porosity of the polymer matrices, as expected. At the same time, the range of wall thickness is reduced (Figure 3(a1–a4), insets) and the mean wall thickness is decreased alongside the polymer volume fraction, which is consistent with increased porosity (Table 1). On the other hand, for Ag-P(NiPAAm/IA) hydrogel nanocomposites, the pore size distribution is narrower, with the majority of pores being less than 50 μm (Figure 3(b1–b4)), while the wall thickness distribution does not show significant changes (Figure 3(b1–b4), insets). The majority of pore wall thicknesses (>90%) were found to be in the range between 2 and 17 μm. However, in the case of nanocomposites, the polymer volume fraction is somewhat higher than the corresponding polymer matrices, which is probably a consequence of the incorporation of AgNPs. Namely, incorporated AgNPs interact with the polymer network and act as additional junction points, i.e., physical crosslinking sites causing the decreasing of porosity [17,33], resulting in the investigated volumes of the samples possessing higher polymer volume fractions. Furthermore, due to instrumental limitations, in the case of nanocomposites, the AgNPs cannot be distinguished from the polymer network, and the volume of the nanoparticles themselves was included in the volume of the polymer, leading to an increase in the polymer volume fraction.

The irregularity of the pore size and the complexity of the pore size distribution can be evaluated using the fractal dimension (FD), defined as the statistical index of the surface complexity of an object, which quantifies how that object’s surface fills 3D space [31]. The less-complex porous structure is characterized by the FD up to 2, and higher values denote a more complex porous structure that offers more space filling [34]. The P(NiPAAm/IA) hydrogels have fractal dimensions between 2.66 and 2.78, whereas these values for the Ag-P(NiPAAm/IA) hydrogel nanocomposites are in the range of 2.77–2.81 (Table 1), indicating that the investigated systems possess a highly complex pore structure distribution. Further insight into the microstructures of crosslinked systems can be achieved by evaluation of the structure model index (SMI), which allows quantification of the characteristic form of a polymer network in terms of the amount of “plates” and “rods” that make up its structure. The SMI values of 0 and 3 are characteristics of perfect plates and perfect rods, respectively [35]. For the investigated samples, the SMI is in the range of 1.46–2.13 (Table 1), indicating that the polymer network contains both plate-like and rod-like structures.

### 3.2. Characterization of AgNPs

Taking into account that exposure of the human body to the influence of nanoparticles is becoming increasingly widespread, it is necessary to perform nanoparticle characterization before their use. The physicochemical characteristics of nanoparticles, such as their size, shape, number of atoms per NP, density and molar concentration in the sample, theoretical surface area, and particle solubility, are essential for their biomedical application and understanding their effects on biological systems [21,36,37].

The optical properties of AgNPs incorporated into P(NiPAAm/IA) hydrogels were investigated by UV-Vis spectroscopy. As presented in our previous study, all obtained UV-Vis absorption spectra showed the characteristic surface plasmon resonance (SPR) bands, with the maxima in the range of 398–411 nm, due to formation of spherical AgNPs. The particle radii (*r_exp_*), calculated using the quasi-static approximation of the Mie theory, are in the range from 4.08 nm to 8.14 nm (Table 2) [17]. Considering these values for AgNPs radii, and assuming that the bulk density of silver (*ρ_Ag_*) is 5.86 × 10^22^ atoms/cm^3^, it is possible to determine the average number of atoms per spherical NP (*N_av_*) in the metallic phase and the NP density in the sample (*D_NPs_*) [38]. The average number of atoms per AgNP was calculated as:

(3) $$N_{av}=\rho_{Ag}{\;V}_{sf},$$

where *V_sf_* is the volume of the nanosphere, while the density of AgNPs in the sample was estimated by dividing the total amount of Ag atoms in the sample volume (*N_tot_*) by the *N_av_* (*N_tot_* is equivalent to the amount of silver in the sample obtained by argon-stabilized arc emission spectrometry). Additionally, the molar concentration of AgNPs in the sample was determined by the relation:

(4) $$C_{NPs}=N_{tot}/N_{av}\;V{\;N}_{A},$$

where *V* is the volume of the reaction solution and *N_A_* is the Avogadro’s constant [21,36,39]. Furthermore, the theoretical surface area (*S.A.*) and solubility of the AgNPs (*S_r_*) were calculated. The theoretical surface area was calculated using:

(5) $$S.A.=6/2\;r_{exp}\;\rho ,$$

where *ρ* is the theoretical density of silver (10.5 g/cm^3^) [21,36,40]. The solubility of NPs is a function of their size, and can be determined by a modified form of the Kelvin equation (Ostwald–Freundlich relation) as:

(6) $$S_{r}=S_{bulk}\;exp\left(2\;\gamma \;V_{m}/R\;T\;r_{exp}\right),$$

where *S_bulk_* is the solubility of silver with a flat surface (0.009 mg/L), *γ* is the surface tension of the particles (1 J/m^2^), *V_m_* is the molar volume of the particles, *R* is the universal gas constant, *T* is the temperature, and *r_exp_* is the experimental value of the radius [21,41]. As can be seen from the values of the AgNP parameters presented in Table 2, the smallest AgNPs possess the highest density and molar concentration in the sample, as well as the highest theoretical surface area and solubility, while increasing the AgNPs radii leads a to significant decrease in those parameters, as expected [21].

**Figure 4:**
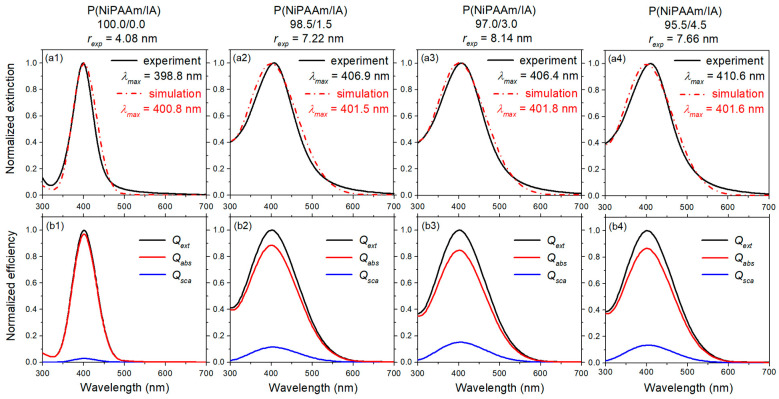
Extinction spectra of Ag-P(NiPAAm/IA) hydrogel nanocomposites: comparison of normalized extinction spectra obtained by experiment and by MiePlot simulation (**a1**–**a4**) and normalized efficiency of MiePlot simulated extinction spectra for AgNPs (**b1**–**b4**): *Q_ext_*—extinction efficiency, *Q_abs_*—absorption efficiency, *Q_sca_*—scattering efficiency.

It is well known that the interaction of light with metal nanospheres results in the absorption and scattering of incident plane waves, with the total energy loss of incident light being equal to the sum of the absorbed and scattered energy [38]. Experimentally obtained spectra are frequently labeled as absorption spectra; however, what is actually being measured are extinction spectra, which are the sum of both the absorption and scattering. These two processes are fundamentally different, and their contribution to the extinction spectra can be a limiting factor for the application of NPs in various fields. For small NPs (*d* < 20 nm), the dominant process is absorption, and for larger ones (*d* > 40 nm) it is scattering, while, for intermediate sizes, these processes can be of a similar order of magnitude [42,43,44]. To evaluate the extinction spectra of Ag-(PNiPAAm/IA) hydrogel nanocomposites, as well as the relative contributions of absorption and scattering, the program “MiePlot v4.6.21” [45] was used. The algorithm of this program is based on Mie’s theory and can calculate the cross sections and/or efficiencies of extinction, absorption, and scattering for spherical NPs as a function of wavelength. Taking into account the experimentally obtained values for the AgNPs’ radii (Table 2), the corresponding theoretical extinction spectra were simulated and presented in Figure 4(a1–a4). A good agreement between the experimentally and theoretically obtained extinction spectra is evident, and the best match of the SPR band position is observed for the smallest AgNPs (Figure 4(a1)). For the larger AgNPs, the positions of the SPR bands in the experimental spectra are red-shifted for 4.6 nm to 9 nm in comparison with the simulated ones. According to the literature, this small red-shift is frequently observed and often associated with inaccurately determined NP diameters/distribution or shape imperfection of the NPs [42]. Namely, the theory considers an ideal nanosphere, but in the experimentally obtained samples, the presence of non-spherical or imperfectly spherical NPs is mostly impossible to avoid, which is confirmed by SEM analysis. As already mentioned, Mie’s theory enables the calculation of the efficiencies of extinction, absorption, and scattering processes during the interaction of the AgNPs with incident light. As can be seen from Figure 4(b1–b4), the simulation confirmed that the absorption is dominant over the scattering for small AgNPs. The simulation results also show that the contribution of scattering in the extinction spectra increases with an increasing size of the AgNPs, from 2.91% for the smallest NPs (*r_exp_* = 4.08 nm) up to 15.13% for the biggest NPs (*r_exp_* = 8.14 nm).

**Figure 5:**
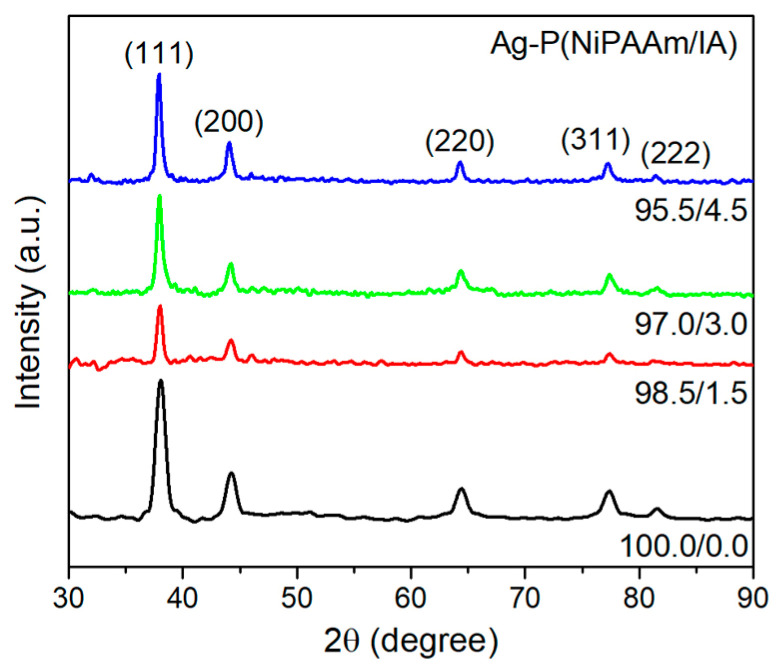
XRD patterns of Ag-P(NiPAAm/IA) xerogel nanocomposites.

Furthermore, the microstructural analysis of AgNPs incorporated into the crosslinked polymer matrix and the ability of the P(NiPAAm/IA) network to protect and stabilize them from coalescence and growth were examined by XRD measurements. The XRD patterns of the investigated nanocomposites (Figure 5) are consistent with the face-centered cubic (*fcc*) crystal structure of bulk metallic silver (JCPDS File No.89-3722). The diffraction maxima at the 2*θ* angle positions of 38.0°, 44.2°, 64.4°, 77.4°, and 81.6° correspond to the Bragg reflections from the crystal planes: (111), (200), (220), (311), and (222). The absence of silver oxide diffraction maxima indicates the existence of pure silver, while the high intensity of the (111) peak indicates a high degree of crystallinity [22,23].

The crystalline domain sizes of the AgNPs were estimated using Scherrer’s equation:

(7) $$D_{Sch}=k_{c}\;\lambda /\beta \cos\theta ,$$

where *k_c_* is the constant for the cubic structure (0.9), *λ* is the X-ray wavelength (0.1541 nm), *β* is the half-width of the diffraction maximum (FWHM), and *θ* is the diffraction angle. Moreover, the interplanar distance (*d*), lattice parameter (*a*), and dislocation density (*δ_D_*) were determined using Equations (8)–(10), respectively, to perform a microstructural analysis of the incorporated AgNPs and to evaluate the influence of the polymer matrix on their microstructural characteristics [22,23,46,47]:

(8) d=n λ/2 sinθ,

(9) $$a=d{\;\left(h^{2}+k^{2}+l^{2}\right)}^{1/2},$$

(10) $$\delta_{D}=15\;\beta \;cos\theta /4\;a\;D_{Sch},$$

where *n* is the diffraction order, and *h*, *k*, and *l* are each the Miller indices of a certain crystallographic plane. The lattice dislocation is a measure of the crystal deformation, a parameter that takes into account the imperfections of nanocrystals within the polymer network. The incorporated AgNPs may be subjected to compression or stretching, depending on the properties of the polymer network. Under the assumption that the crystalline domain size and deformation are interdependent parameters, the Williamson–Hall equation can be used to determine the lattice strain (*ε*) from the slope of the plot *β*cos*θ* vs. 4sin*θ* [48,49]:

(11) $$\beta cos\theta =\frac{k_{c}\;\lambda }{D_{WH}}+4\;\varepsilon \;sin\theta ,$$

In addition, the modified Williamson–Hall method (Equation (12)) can be used to calculate the lattice stress that arises in crystallites during the growth process [48,49]:

(12) $$\beta cos\theta =\frac{k_{c}\;\lambda }{D_{WHM}}+\frac{4\;\sigma \;sin\theta }{Y}$$

where Y is the theoretical value of the Jung modulus of elasticity for silver (83 GPa), while the value of the lattice stress (*σ*) was obtained from the slope of the plot *β*cos*θ* vs. 4sin*θ/*Y. The obtained microstructural parameters of the AgNPs are presented in Table 3.

According to the obtained results, the crystalline domain size (*D_Sch_*) of AgNPs is the smallest in the homopolymer network (100.0/0.0) and increases with an increase in IA content, as expected. Namely, the addition of hydrophilic IA into the copolymer network leads to an increase in the porosity and mean pore diameter, and, consequently, to a decrease in the polymer volume fraction (Table 1). The lower polymer content in the network results in the weaker stabilization of the AgNPs and less controlled crystal growth, leading to the formation of slightly larger NPs. The obtained values of *D_Sch_* are in good agreement with the average diameter of the AgNPs estimated by UV-Vis analysis.

Moreover, the polymer content in the network also influences the microstructural parameters of the AgNPs. The values of lattice parameter (*a*) and the interplanar distance (*d*), evaluated on the main diffraction maxima (111), are somewhat higher, but generally, they are in a good agreement with the bulk *fcc* Ag (*d*_0_ (111) is 0.2355 nm and *a*_0_ is 0.4086 nm (JCPDS File No.89-3722)). Compared to the *a*_0_, the values of the lattice parameter increase from 0.1% for the homopolymer network (100.0/0.0) up to 0.5% for the network with the highest content of IA (95.5/4.5), indicating the occurrence of crystallites elongation (existence of tensile deformations). The changes in the lattice parameters of crystalline AgNPs reflect their state of strain and stress, which depends on the formation conditions. In the homopolymer network (100.0/0.0), the crystallites are positively strained and under tensile stress (*ε* > 0 and *σ* > 0), while the addition of IA into the copolymer network induces the opposite trend. In this case, the crystallites are negatively strained and under compression stress (*ε* < 0 and *σ* < 0), and the obtained values slightly decreased with an increasing IA content. The internal stress states of NPs are mainly determined by the surface stresses, elastic anisotropy, and particle geometry. For the NPs incorporated into the matrix of foreign material, the interface stress, which represents the elastic response of the interface between both materials to elastic deformation, should be considered instead of the surface stress. The interface stress is related to the strength of the interaction (adhesion work) of the phases adjacent to the interface [50]. We assume that negative values of lattice strain and lattice stress are correlated with a change in the surrounding medium in which the formation of AgNPs occurs. Namely, the addition of IA into the copolymer network leads to an increase in the number of hydrophilic carbonyl and carboxyl groups, which results in an increase in the electrostatic interaction between the AgNPs and the polymer matrix (metal/matrix interface) and, thus, directly contributes to the interface stress. Finally, the dislocation density (*δ_D_*) was estimated as a parameter that provides information about imperfections in Ag nanocrystals. As can be seen from Table 3, the obtained values decrease with the increase in the AgNPs’ size and the content of IA in the copolymer network. The highest dislocation density was observed for the AgNPs incorporated into the homopolymer network (100.0/0.0). The macroscopic properties of materials can be largely affected by their microstructure. For example, the mechanical strength of materials increases with an increase in their dislocation density, which is confirmed by mechanical analysis of the investigated Ag-P(NiPAAm/IA) hydrogel nanocomposites [17,51]. In addition, the dislocation density is also a parameter that may indicate the potential antibacterial properties of different NPs. Research has shown that NPs with higher values of dislocation density possess better antibacterial activity [23,52].

**Figure 6:**
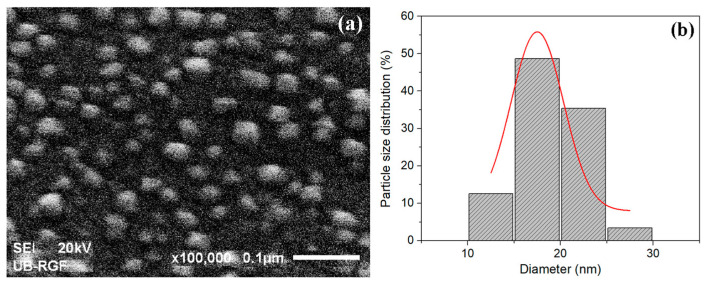
SEM micrograph of AgNPs (sample Ag-P(NiPAAm/IA) 97.0/3.0) (**a**) and AgNP size distribution (**b**). The red line represents the Gaussian fit function of experimental data for particle size distribution.

To corroborate the average AgNP size obtained by UV-Vis spectroscopy and XRD measurement, the Ag-(PNiPAAm/IA) 97.0/3.0 xerogel nanocomposite was observed by SEM, as an example. As can be seen from the SEM micrograph (Figure 6a), a rather uniform distribution of the AgNPs was observed, with a size range from 10 nm to 30 nm, and an average diameter of around 17.5 nm (Figure 6b). The synthesized AgNPs are not perfect spheres, but most of them have spherically symmetric shapes, with the size distribution having around the same average value as that determined by the other two methods.

### 3.3. Hydrogels Swelling Behavior in Simulated Skin Treatment Condition

Considering the potential biomedical application of the investigated systems as wound dressings, the fundamental properties necessary for their physicochemical characterization are their swelling capacity and swelling kinetic parameters. To mimic the conditions of a physiological environment, the swelling of P(NiPAAm/IA) hydrogels and Ag-P(NiPAAm/IA) hydrogel nanocomposites was investigated at 37 °C in two different mediums, distilled water and SBF. The swelling kinetics of the samples were monitored at proper time intervals until they reached an equilibrium state.

The structural factors of the polymer networks and the properties of the swelling medium affect the swelling characteristics of hydrogels. Therefore, the swelling of hydrogels is a process that can be controlled and modified by the polymer concentration, copolymers ratio, and crosslinking degree, as well as by the environmental conditions where the application was planned. Spasojević et al. [17] have shown that Ag-P(NiPAAm/IA) hydrogel nanocomposites possess both thermo- and pH responsiveness, with the swelling capacity being influenced by the network composition and the presence of AgNPs. When increasing the IA content in the copolymer matrix and the pH value of the swelling medium, the swelling capacity of the investigated systems increased, while the incorporation of AgNPs led to a decrease in the swelling capacity of the hydrogel nanocomposites compared to that of the pure P(NiPAAm/IA) hydrogels. This class of “smart” hydrogels has been widely used for biomedical application due to their resemblance to biological tissues and their swelling/shrinking response to small changes in pH or temperature as an environmental trigger [53,54].

**Figure 7:**
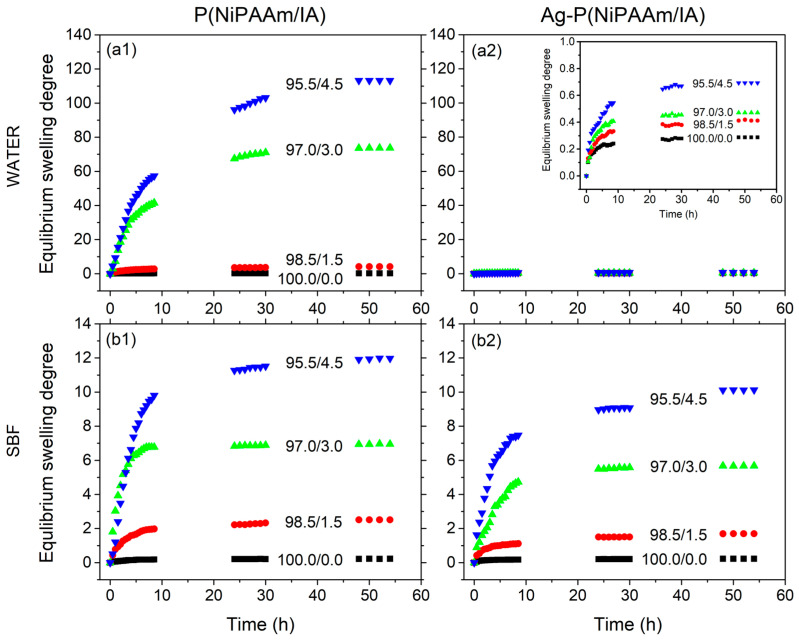
Swelling curves of P(NiPAAm/IA) hydrogels (**a1**,**b1**) and Ag-P(NiPAAm/IA) hydrogel nanocomposites (**a2**,**b2**) in distilled water (**up**) and SBF (**down**) at 37 °C.

It is well known that PNiPAAm hydrogels have a volume phase transition temperature (VPTT) of around 32 °C as a consequence of a sharp coil–globule transition that occurs as a response to environmental temperature changes [17]. The values of VPTT may be changed by copolymerization with hydrophilic ionic comonomers (for example IA), resulting in a copolymer network with both thermo- and pH sensitivity [54]. In previous work, it was shown that the VPTT of P(NiPAAm/IA) hydrogels increases with an increasing IA content, while the incorporation of AgNPs leads to a small decrease in the VPTT values compared to those of the pure copolymer network [17]. Bearing in mind the abovementioned, it is evident that, under the experimental condition (37 °C), almost all investigated samples passed through the phase transition point where the PNiPAAm component was in its shrunken state, giving significantly lower values of the equilibrium swelling degree (*SD_eq_*) (Table 4) in comparison with the values obtained in distilled water at 25 °C [17]. The swelling curves of the P(NiPAAm/IA) hydrogels and Ag-P(NiPAAm/IA) hydrogel nanocomposites in distilled water and SBF at 37 °C are presented in Figure 7.

The P(NiPAAm/IA) hydrogels’ swelling process is dominated by the thermo-sensitivity of the PNiPAAm component, while, in the case of Ag-P(NiPAAm/IA) hydrogel nanocomposites, two effects must be distinguished: first is the contraction of the thermosensitive component, and the second is the influence of the incorporated AgNPs. This influence is probably mostly expressed through the electrostatic interactions between AgNPs and the hydrophilic carbonyl and carboxyl groups of IA, which act as additional physical crosslinking and thus reduce the swelling capacity to a minimum level (Figure 7(a2) inset). Moreover, the swelling process was monitored in SBF, which simulated the conditions in human blood plasma, to investigate the influence of existing ions on the swelling capacity. As can be seen from Figure 7 and Table 4, the P(NiPAAm/IA) hydrogels have a lower swelling capacity, while the Ag-P(NiPAAm/IA) hydrogel nanocomposites have a higher swelling capacity in SBF, in comparison to in the distilled water. We assume that this is caused by the presence of different ions in SBF [24] that may interact both with the functional groups in the copolymer network and with the incorporated AgNPs.

To analyze the swelling process, it is needed to determine the mechanism of fluid transport into the polymer network. The swelling process can be described by applying the following equation:

(13) $$\frac{SD}{{SD}_{eq}}=k\;t^{n},$$

where *k* is the kinetic rate constant, *t* is the time, and *n* is a diffusion exponent that determines the mechanism of fluid transport into the polymer matrix. The logarithmic form of Equation (13) gives a linear dependence at the initial stage of swelling (*SD/SD_eq_* ≤ 0.6), and the values of *n* can be determined from the slope. Generally, there are three diffusion mechanism models: (i) Fick or type I diffusion (*n* ≤ 0.5), where the diffusion process is dominant, (ii) non-Fick or anomalous diffusion (0.5 < *n* < 1), when the effects of the diffusion and relaxation of polymer chains are comparable, and (iii) type II diffusion (*n* = 1), when the relaxation process of polymer chains is dominant [55]. As can be seen from Table 4, most of the investigated samples show non-Fick diffusion, with diffusion exponents in the range from 0.51 up to 0.88 in both swelling media, indicating that fluid diffusion and polymer chains’ relaxation are comparable. An exception is noticeable only for samples with 0.0 wt% and 1.5 wt% of IA during the swelling in distilled water. In these cases, the swelling of the hydrogels is a diffusion-controled process, probably due to them having the highest polymer volume fraction (Table 1) and lowest VPTT (below the experimental conditions) [17].

The fundamental process of diffusion is based on Fick’s law, which describes the macroscopic transport of molecules by a concentration gradient, and enables the determination of the diffusion coefficients (*D*) as:

(14) $$D={\left(k\;\pi \;r^{2}/4\right)}^{1/n}$$

where *r* is the radius of the xerogels [55]. The values of the calculated diffusion coefficients increase with an increase in the *SD_eq_* and IA content in copolymer networks (Table 4), as expected. Moreover, it is noticeable that the incorporation of AgNPs decreased the *D* values compared to those of the pure P(NiPAAm/IA) hydrogels, probably due to the smaller chain flexibility caused by the presence of NPs, which can act as additional junction points, resulting in a lower porosity of the hydrogel nanocomposites (Table 1).

### 3.4. Biomedical Potential of Ag-P(NiPAAm/IA) Hydrogel Nanocomposites

Nowadays, AgNPs have probably become one of the most explored and exploited nanostructures because of their unique physicochemical properties. The widespread and diverse commercial and biomedical applications of AgNPs have increased their potential health hazards that cannot be overlooked due to their uncontrollable use, discharge to the natural environment, and impact on it, as well as potential toxic effects.

Bearing in mind that the investigated Ag-P(NiPAAm/IA) hydrogel nanocomposites can potentially be used as wound dressings and patches, the evaluation of their biomedical potential was performed by monitoring their *in vitro* release of Ag^+^ ions, antimicrobial activity, and cytotoxicity. Considering that a high concentration of silver can cause serious cytotoxicity and genotoxicity effects in human cells, a controlled and sustained, steady supply of Ag^+^ ions from the Ag-P(NiPAAm/IA) hydrogel nanocomposites is one of the most important parameters to examine. The antibacterial potential of hydrogel nanocomposites is mostly influenced by the size and shape of the incorporated AgNPs, as well as by the uncontrolled release of Ag^+^ ions from the polymer matrix. To overcome these issues, the selection of a polymer matrix with good structurally compatible properties is crucial for retaining and controlling the release of silver ions and/or AgNPs [56,57]. In the investigated systems, the AgNPs are embedded in P(NiPAAm/IA) copolymer networks which effectively prevent their releasing in the environment in larger amounts due to interactions between the AgNPs and specific groups in the polymer chains [17].

In general, there is broad agreement that the release of Ag^+^ ions is a major pathway for the biological activity of AgNPs. Previous studies have shown that most of the Ag^+^ ions are formed by oxidation of the zerovalent metallic NPs. Because the release of Ag^+^ ions from AgNPs’ surfaces is primarily a heterogeneous oxidation reaction involving the cooperative effects of dissolved O_2_ and protons, this suggests surface area dependency and the possibility to control the Ag^+^ ions’ release by controlling the NP sizes [21,58,59,60].

**Figure 8:**
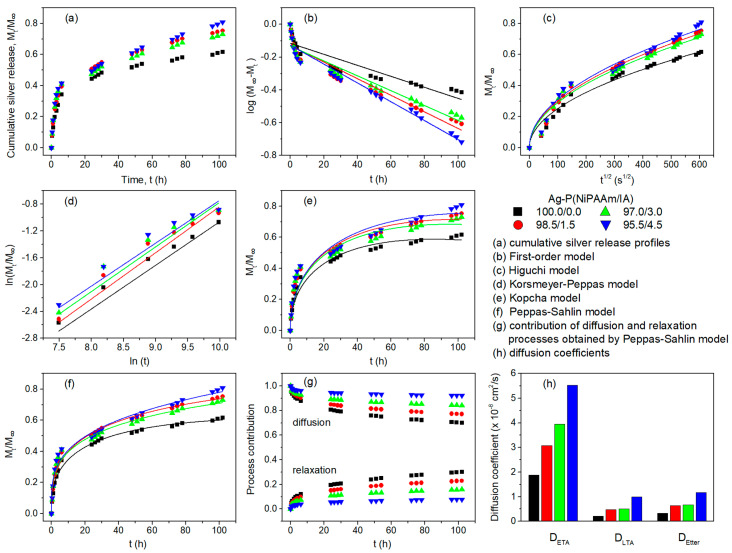
Cumulative silver release profiles (**a**), mathematical modeling (**b**–**g**), and diffusion coefficients (**h**) for Ag-P(NiPAAm/IA) hydrogel nanocomposites.

To simulate physiological conditions, the release of Ag^+^ ions from Ag-P(NiPAAm/IA) hydrogel nanocomposites was monitored in phosphate buffer solution (PBS) at 37 °C, and the *in vitro* cumulative release profiles are presented at Figure 8a. As can be seen, after 4 days of silver release, the Ag-P(NiPAAm/IA) (100.0/0.0) hydrogel nanocomposite released the smallest amount of initial concentration of silver (59.9%), whereas the samples with 1.5 wt%, 3.0 wt%, and 4.5 wt% of IA in their copolymer network released 73.8%, 71.1%, and 78.2% of an initial concentration of silver, respectively. According to the physicochemical parameters of AgNPs (Table 2), the smallest NPs are embedded in the homopolymer network (100.0/0.0) and possess the highest values of theoretical surface area and solubility. Consequently, it would be expected that these systems release the highest amount of silver, compared to the initial concentration, but the opposite trend is evident. This is probably due to the highest polymer volume fraction and the lowest porosity, in the case of homopolymer network (Table 1), resulting in the greatest stability of the AgNPs. Moreover, under the given experimental condition (PBS, pH = 7.4 at 37 °C), the thermosensitive PNiPAAm component is in the shrunken state, while the addition of IA (pH-sensitive component) leads to an increase in the swelling capacity [17] and thus enables the facilitated dissolution of AgNPs and diffusion of Ag^+^ ions from the polymer networks.

As can be seen from the release profiles (Figure 8a), all investigated Ag-P(NiPAAm/IA) hydrogel nanocomposites release more than 50% of silver, regarding their cumulative release concentration during 4 days of investigation, in the first 6 h of immersion in PBS, followed by a sustained slower release. Considering the desired applications of the investigated systems as wound dressings, the initial burst release of a certain amount of silver is useful because it prevents bacteria adherence and biofilm formation, whilst the successive slower release ensures the sterility of the wound and the dressing for extended durations of use [61,62,63]. To achieve the maximum effect of wound dressings, it is advisable to replace the hydrogel dressing with a new one around the time when the release profile reaches a plateau of slower release [7,57,64]. In the case of the investigated Ag-P(NiPAAm/IA) hydrogel nanocomposites, this replacement period would be approximately 2–3 days, to ensure a steady supply of the wound with the antimicrobial agent and to prevent infection.

The release of Ag^+^ ions from hydrogel nanocomposites can be controlled by diffusion or polymer chains’ relaxation, as well as by the combination of these two effects. To evaluate the silver release kinetics of the tested materials, the experimental data were fitted and compared with several commonly used kinetics models of the drug release process, which are described by Equations (15)–(21) [2,65,66,67].

(15) $$\mathrm{First}\text{-}\mathrm{order}\;\mathrm{model}:\;logM_{t}=logM_{0}-k_{1}\;t/2.303$$

where *M_t_* is the amount of silver released in time *t*, *M*_0_ is the amount of silver released in time *t* = 0 (*M*_0_ = 0), and *k*_1_ is the first-order rate constant. In this case, the drug released at each time is proportional to the residual drug inside the dosage form.

(16) $$\mathrm{Higuchi}\;\mathrm{model}:\;\frac{M_{t}}{M_{\infty }}=k_{H}\;t^{1/2}$$

where *M_t_/M_∞_* is the fraction of silver released at each time point (*M_∞_* is the initial amount of silver in hydrogel nanocomposites) and *k_H_* is the Higuchi dissolution rate constant. This is probably the most often-used model, which describes the release of drugs from an insoluble matrix as a square root of a time-dependent process based on Fick’s law.

(17) $$\mathrm{Korsmeyer}–\mathrm{Peppas}\;\mathrm{model}:\;\frac{M_{t}}{M_{\infty }}=k_{KP}\;t^{n}$$

where *k_KP_* is the Korsmeyer–Peppas constant, dependent on the polymer network properties, while *n* is the diffusional exponent, which indicates the transport mechanism during the release. This model describes the drug release from a polymeric system by a simple and effective power-law approach, and it is applicable to the first 60% of drug release (*M_t_/M_∞_* < 0.6).

(18) $$\mathrm{Kopcha}\;\mathrm{model}:\;\frac{M_{t}}{M_{\infty }}=A\;t^{1/2}+B\;t$$

where *A* and *B* are the Kopcha constants, indicating the dominant process during the release (*A* is the diffusional constant and *B* is the relaxation constant). This model is used to determine the relative contribution of diffusion and polymer chains’ relaxation to the process of drug release from the polymer matrix. If the A value is much greater than the B value, the drug release will follow the diffusion mechanism, while if the B value is higher than the A value, the dominant drug release mechanism is the polymer relaxation mechanism.

(19) $$\mathrm{Peppas}–\mathrm{Sahlin}\;\mathrm{model}:\;\frac{M_{t}}{M_{\infty }}=K_{1}\;t^{m}+K_{2}\;t^{2m}$$

where *K*_1_ and *K*_2_ are the diffusion and relaxation rate constants, respectively, while *m* is the diffusion exponent. This model enables the quantification of the approximate contribution and coupled effect of the diffusion and polymer relaxation mechanisms of an anomalous drug release process. The first term on the right-hand side of Equation (19) represents the Fickian diffusional contribution (*F*), whereas the second term is the contribution of the polymeric system relaxation (*R*). The ratio of both contributions (*R*/*F*) can be calculated using Equation (20), while the amount of drug released due to the diffusion mechanism (*F*) can be determined by Equation (21):

(20) $$\frac{R}{F}=\frac{K_{2}}{K_{1}}\;\;t^{m}$$

(21) $$F=1/\left(1+\frac{K_{2}}{K_{1}}\;t^{m}\right)$$

The mathematical modeling of the silver release was performed using the abovementioned equations to determine the best fit with the experimental data (Figure 8b–f) by evaluating the values of the correlation coefficients (*R*^2^). The obtained fitting parameters are summarized in Table 5.

The curvilinear nature of the cumulative silver release profiles is obvious, indicating that the rate of silver release from the Ag-P(NiPAAm/IA) hydrogel nanocomposites is not constant over time, i.e., it does not follow the zero-order kinetics model (not presented), which is supported by the lowest values of *R*^2^ being in the range from 0.736 to 0.813 [68]. In the case of first-order kinetics, when soluble active agents are incorporated into a porous matrix, the amount of drug released must be proportional to the amount of drug remaining in the matrix. Therefore, the amount of drug released should decrease linearly with time [69]. According to Figure 8b and the low *R*^2^ values (0.833–0.949) compared to those of other models, it can be concluded that the silver release from the investigated hydrogel nanocomposites does not follow the first-order kinetics model either. On the other hand, the high values of *R*^2^ (0.975–0.979) for the Higuchi model indicate that it shows a good fit with the experimental data (Figure 8c). The Higuchi model can be applied to drug release systems in which the drug delivery arises through both dissolution and diffusion mechanisms [70]. The formation of a concentration gradient due to the dissolution of AgNPs is the main trigger for the diffusion of Ag^+^ ions from the polymer matrix into the surrounding medium, and the Higuchi dissolution rate constant (*k_H_*) values are higher for the systems with enhanced swellability (higher IA content), as expected. To determine which type of diffusion is dominant, the experimental data were fitted to the Korsmeyer–Peppas model (Figure 8d). In the case of the cylindrical samples, n ≤ 0.45 corresponds to a Fickian diffusion, 0.45 < n < 0.89 to non-Fickian diffusion, and n ≥ 0.89 to swelling-controlled release (Case II transport) [69,71]. For the examined Ag-P(NiPAAm/IA) hydrogel nanocomposites, the diffusional exponent (n = 0.58–0.63) showed that the silver release mechanism is non-Fickian or anomalous diffusion (*R*^2^ = 0.952–0.990), governed by both diffusion and polymer chain relaxation. However, the Korsmeyer–Peppas model cannot distinguish the predominant mechanism during the silver release. Thus, the Kopcha (Figure 8e) and Peppas–Sahlin (Figure 8f) models were employed to gain a deeper insight into the release process. The Kopcha model shows a good fit with the experimental data (*R*^2^ = 0.930–0.976) and was used to quantify the relative contributions of diffusion and polymer relaxation to the silver release. The obtained values clearly show that the diffusional constants (*A*) are far greater than the relaxation constants (*B*), indicating that the Ag^+^ ion release from the hydrogel nanocomposites predominantly occurred by a Fickian diffusion process [21]. Furthermore, the Peppas–Sahlin model was used to approximate the contribution of each mechanism, diffusion and relaxation, in an anomalous silver release process. As can be seen from the results, the relaxation rate constants (*K*_2_) have much lower values than the diffusion rate constants (*K*_1_), suggesting that the polymer chain relaxation has an insignificant effect compared to the diffusion [2]. With the increase in IA content in the polymer network, the rate constant values increase because of the greater porosity and higher swelling capacity of the network, and this enables the easier and faster swelling of polymer matrix, as well as the dissolution and diffusion of silver from the hydrogel nanocomposites. Using the parameters obtained by the Peppas–Sahlin model, the diffusional contribution (*F*) and relaxation contribution (*R*) during the silver release were determined by applying Equations (20) and (21), and the results are depicted in Figure 8g. It is evident that the composition of the polymer matrix does not have a significant effect on the silver release mechanism. For all Ag-P(NiPAAm/IA) hydrogel nanocomposites, the predominant mechanism is Fickian diffusion, with a small contribution from chain relaxation, especially at the beginning of the process (initial burst release). As the length of the study time increased, the diffusional contribution decreased and was inversely proportional to the relaxation contribution but continued as the main mechanism [72]. Among the applied models, the Peppas–Sahlin model exhibited the best fit with the experimental data, exhibiting the highest correlation coefficient values (*R*^2^ = 0.976–0.990).

Finally, the diffusion coefficients of silver ions from the polymer matrix were determined by the models based on the solutions of Fick’s law with three approximations: the early-time approximation (Equation (22)), the late-time approximation (Equation (23)), and the Etters approximation (Equation (24)) [17,21,73]:

(22) $$\frac{M_{t}}{M_{\infty }}=4{\left(\frac{D_{ETA\;\;}\;t}{\pi {\;\delta }^{2}}\right)}^{1/2}$$

(23) $$\frac{M_{t}}{M_{\infty }}=1-\frac{8}{\pi^{2}}exp\left(\frac{{-D}_{LTA}\;\pi^{2}\;t\;}{\delta^{2}}\right)$$

(24) $$\frac{M_{t}}{M_{\infty }}={\left[1-exp\left({-K\left(\frac{D_{Etter}\;t}{\delta^{2}}\right)}^{a}\right)\right]}^{1/b}$$

where *D_ETA_* is the diffusion coefficient of silver for the early stage of releasing (*M_t_*/*M_∞_* < 0.6), *D_LTA_* is the diffusion coefficient of silver for the late stage of releasing (*M_t_*/*M_∞_* > 0.6), *D_Etter_* is the diffusion coefficient of silver for the entire range of releasing (0 < *M_t_*/*M_∞_* < 1), *t* is the time, and *δ* is the thickness of the xerogel. For the Etters model, the constants are = 1.3390, b = 2.6001, and K = 10.5449 [73].

From the results presented in Figure 8h, it can be seen that all three diffusion coefficients of silver increase with the increase in IA content in the polymer matrix. Such behavior is expected according to the physicochemical properties of Ag-P(NiPAAm/IA) hydrogel nanocomposites presented in Table 4. Namely, with an increase in the IA content in the polymer matrix, the swelling capacity of hydrogel nanocomposites and the diffusion coefficients of the medium into the hydrogel nanocomposites increase, thus enabling the easier and faster dissolution of silver. Generally, the values of *D_ETA_* are the highest (from 1.88 × 10^−8^ to 5.54 × 10^−8^ cm^2^/s), while the values of *D_LTA_* are the lowest (from 0.21 × 10^−8^ to 0.99 × 10^−8^ cm^2^/s), which is in good agreement with the initial burst release at the early stage and steady slower release in the middle and latter stages of releasing, respectively. The values of *D_Etter_* are between those of *D_ETA_* and *D_LTA_* (from 0.33 × 10^−8^ to 1.16 × 10^−8^ cm^2^/s), indicating that the Etters approximation “smoothed” the transition in the profile between the early and late stages of silver release [74]. Nevertheless, among the three applied approximations, the Etters approximation showed the worst fit with the experimental results, obtaining the lowest correlation coefficients (*R*^2^), in the range of 0.901–0.990. On the other hand, the early-time and late-time approximations show a better fit to the experimental results, with *R*^2^ values within the range of 0.971–0.995 and 0.981–0.998, respectively.

It is generally known that the skin and soft tissues are the most vulnerable to infection in humans, and that infections are thus particularly prevalent in wounds. The Gram-positive bacteria (*S. aureus* and *S. pyogenes*) are the dominant pathogens in the early stages of infection, while the Gram-negative bacteria (*E. coli*) can be involved in later stages, usually in chronic wounds. To support the body’s immune system in the fight against infection, it is recommended to use anti-infective wound dressings. Nowadays, AgNPs, as an alternative to traditional antibiotics, are some of the most effective antimicrobial agents that have been widely used in wound dressings [75]. In our previous study, it was demonstrated that an Ag-P(NiPAAm/IA) 95.5/4.5 hydrogel nanocomposite, as a test sample, possessed antibacterial properties [17]. In this study, we take a step forward and examine the antimicrobial properties of a series of samples. The antibacterial tests were performed on a series of Ag-P(NiPAAm/IA) hydrogel nanocomposites using the disk diffusion method and optical density method against *E. coli* and *S. aureus* bacteria.

**Figure 9:**
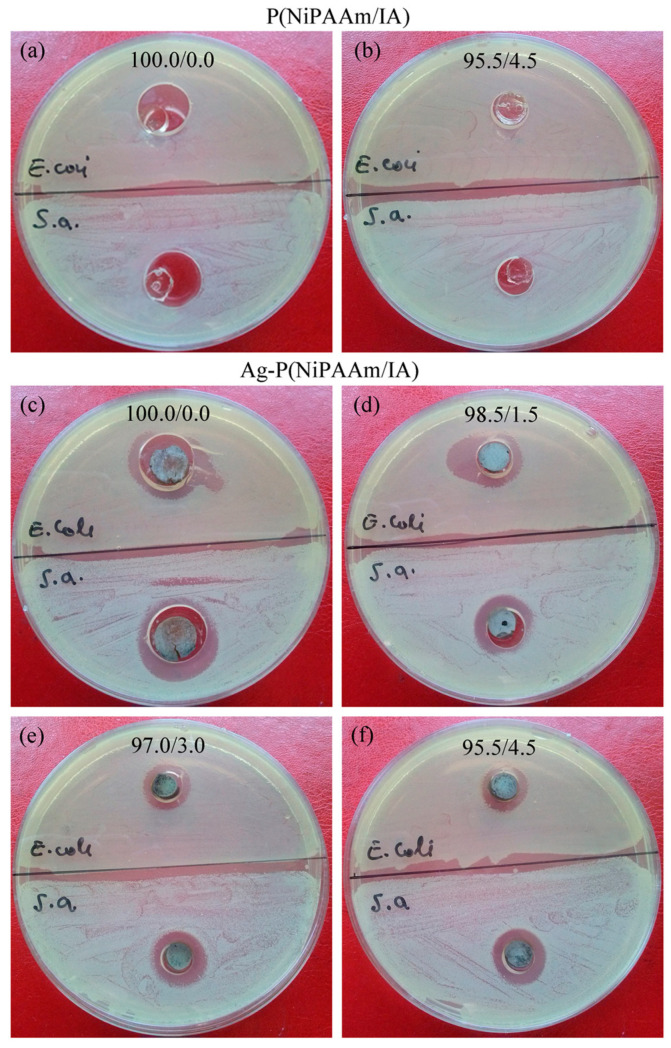
Antibacterial activity of P(NiPAAm/IA) hydrogels (**a**,**b**) and Ag-P(NiPAAm/IA) hydrogel nanocomposites (**c**–**f**) against *Escherichia coli* and *Staphylococcus aureus* after 24 h of incubation at 37 °C [(**b**,**f**): reproduced from reference [17] with permission from Elsevier].

The results for the antimicrobial activity obtained by the disk diffusion method after 24 h of incubation at 37 °C are presented in Figure 9. For the purposes of this study, all Ag-P(NiPAAm/IA) hydrogel nanocomposites were synthesized to contain silver in a concentration of about 250 ± 20 ppm. According to the literature, these and even higher silver concentrations have been used for topical application for scarless wound healing and the treatment of severe forms of infection in diabetology [15,76].

In the case of the polymer matrix, the two hydrogels that were tested, P(NiPAAm/IA) 100.0/0.0 and P(NiPAAm/IA) 95.5/4.5, i.e., a homopolymer network and a polymer network, with the highest contents of IA. It is evident that none of these samples show antimicrobial activity (Figure 9a,b). On the other hand, the Ag-P(NiPAAm/IA) hydrogel nanocomposites exhibit antibacterial activity against both Gram-negative and Gram-positive bacteria (Figure 9c–f). The formed inhibition zone is not quite uniform, and it is the largest for the homopolymer Ag-P(NiPAAm/IA) 100.0/0.0 hydrogel nanocomposite, with a diameter of around 16.5 mm and 18.8 mm for *E. coli* and *S. aureus*, respectively. The addition of IA into the polymer matrix leads to a slight decrease in the diameters of the inhibition zones (≈13–14 mm for *E. coli* and ≈14–15 mm for *S. aureus*), which is in good correlation with the increase in the AgNPs’ size (Table 2). These findings show that smaller AgNPs possess stronger antimicrobial activity, which is in agreement with the literature [77]. Moreover, the enhanced antimicrobial activity in the case of the smallest AgNPs in the Ag-P(NiPAAm/IA) 100.0/0.0 hydrogel nanocomposite was expected due to it exhibiting the highest value of dislocation density (Table 3) [50].

**Figure 10:**
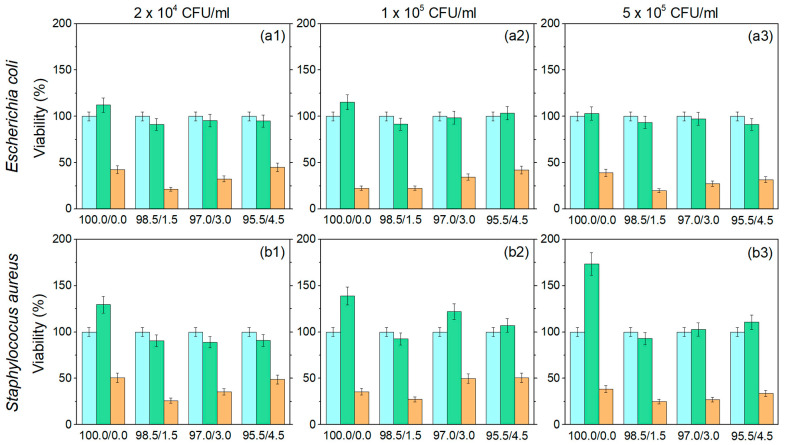
Viability of *Escherichia coli* (**a1**–**a3**) and *Staphylococcus aureus* (**b1**–**b3**) in the presence of P(NiPAAm/IA) hydrogels (green) and Ag-P(NiPAAm/IA) hydrogel nanocomposites (orange) after 24 h of incubation at 37 °C. Control samples are untreated bacterial cultures (blue).

Optimization of the Ag^+^ ions delivery process and fine tuning of the antibacterial properties between the minimum inhibitory concentration (MIC) and minimum bactericidal concentration (MBC) is the most important request for biological applications. The typical MIC and MBC against standard reference cultures as well as multidrug-resistant organisms are 0.78–6.25 ppm and 12.5 ppm, respectively [15]. To meet these requirements, Ag-P(NiPAAm/IA) hydrogel nanocomposites with a silver concentration of around 10 ± 1 ppm were prepared and their antimicrobial activity was analyzed using the optical density method. The percentage of surviving bacteria (*E. coli* and *S. aureus*) in contact with the investigated samples after 24 h of incubation at 37 °C is presented in Figure 10. Three different concentrations of each strain of bacteria were analyzed, and the presented results are the mean value of three independent measurements.

The presence of P(NiPAAm/IA) hydrogels (green columns) generally does not affect the viability of the investigated strain of bacteria. The viability of *E. coli* is slightly reduced in most cases, with a slight tendency of proliferation for the homopolymer matrix (100.0/0.0). Conversely, the proliferation of *S. aureus* is more pronounced, especially in the case of the homopolymer matrix. On the other hand, all Ag-P(NiPAAm/IA) hydrogel nanocomposites (orange columns) showed very good antibacterial activity against both strains of bacteria after 24 h of exposure. In the case of *E. coli*, the number of surviving bacteria was reduced by 55% up to 80% (Figure 10(a1–a3)), while for *S. aureus*, the number of bacteria was reduced by 49% up to 75% (Figure 10(b1–b3)). The slightly better antibacterial activity for *E. coli* in comparison to that for *S. aureus* could be related to differences in the cell wall structures of these bacteria. Namely, the cell walls of Gram-negative bacteria are composed of a thick lipopolysaccharide layer which surrounds a thin layer of peptidoglycans, so the binding of AgNPs or released silver ions to anionic surface domains may enhance their toxicity. In contrast, Gram-positive bacteria have a thin outer lipopolysaccharide layer and a thick inner peptidoglycan layer, which reduces the chances of AgNPs or released silver ions penetrating into the cells and thus provides protection from toxicity [78,79]. Moreover, the Ag-P(NiPAAm/IA) hydrogel nanocomposite with the lowest content of IA (98.5/1.5) possesses the strongest bactericidal effect for both Gram-negative and Gram-positive bacteria, for all bacteria concentrations.

The exact mechanism behind the antibacterial activity of AgNPs has not yet been fully understood and defined. One possible mechanism is the continuous release of Ag^+^ ions by AgNPs, which can adhere to the cell wall and cytoplasmic membrane due to their electrostatic attraction and affinity for sulfur proteins. The adhered ions can enhance the cytoplasmic membrane’s permeability, which may lead to the disruption of the bacterial envelope. After the penetration of Ag^+^ ions into the cells, the respiratory enzymes can be deactivated, generating reactive oxygen species (ROS) which can be a major agent responsible for cell membrane disruption and DNA modification. Also, the interaction of Ag^+^ ions with the sulfur and phosphorus of DNA can interrupt DNA replication and cell reproduction, or even result in cell death. Moreover, Ag^+^ ions can inhibit the synthesis of proteins by denaturing ribosomes in the cytoplasm [80,81]. In addition to releasing silver ions, the AgNPs can kill bacteria on their own. They can anchor to the cell surface and accumulate in the pits formed on the cell wall, thus causing cell membrane denaturation. Due to their nano sizes, the AgNPs may also pass through bacterial cell walls and alter the structure of the cell membrane. Denaturation of the cytoplasmic membrane can cause organelle rupture and cell lysis. Furthermore, silver nanoparticles may be involved in bacterial signal transduction, inducing its disruption and resulting in cell apoptosis and the termination of cell proliferation [81,82].

Regardless of the mechanism of action, the obtained results indicate that the investigated Ag-P(NiPAAm/IA) hydrogel nanocomposites possess a very good biocidal effect and the potential to reduce bacterial growth in practical applications. The great advantage of such types of antibacterial systems is their low silver concentration, which makes them safe for wider use, especially in bioapplications.

Although the investigated hydrogel nanocomposites are showing promising bactericidal effects within therapeutically permitted doses, it is necessary to examine their potential negative effects on the surrounding tissue. In order to assess the biocompatibility of a material, it is important to perform an initial screening of the material’s toxicity effect on the tested biological cells, because cytotoxicity is considered an important indicator of cell viability. Therefore, regarding the potential application of these nanocomposites for topical treatment, the effects of Ag-P(NiPAAm/IA) hydrogel nanocomposites on human HaCaT keratinocyte cells were investigated. For this purpose, nanocomposite samples with the same silver concentration (10 ± 1 ppm) as in the antimicrobial activity test (optical density method) were used to evaluate their cytotoxic effect. In addition, the cytotoxicity of P(NiPAAm/IA) hydrogels was also monitored.

**Figure 11:**
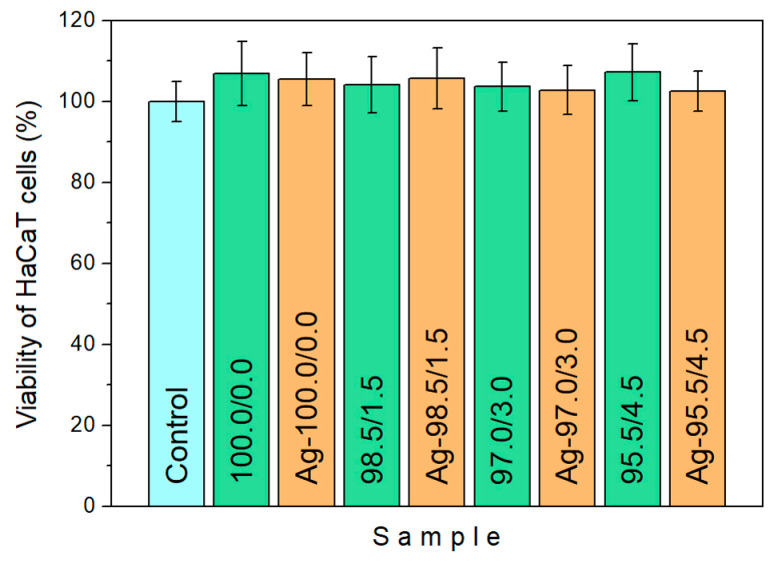
Viability of human keratinocytes HaCaT cell line exposed to P(NiPAAm/IA) hydrogels (green) and Ag-P(NiPAAm/IA) hydrogel nanocomposites (orange) after 24 h of treatment. The control sample is an untreated HaCaT cell line (blue).

The results for human keratinocytes HaCaT cell viability, obtained by the crystal violet test, are given in Figure 11. It is clearly noticeable that neither the P(NiPAAm/IA) hydrogels nor the Ag-P(NiPAAm/IA) hydrogel nanocomposites exhibit a cytotoxic effect. Moreover, the proliferation of HaCaT cells up to 7.2% for the pure polymer matrices and up to 5.7% for the nanocomposite samples was observed, compared to those for the untreated HaCaT cells used as control.

Wound healing is a complex multistep process that involves integrating the activities of different cell and tissue types. During the early stage of wound healing, re-epithelialization and wound contraction are two crucial processes that occur. Re-epithelialization is driven by the migration and proliferation of keratinocytes in the epidermal layer of skin from the wound edge, whereas wound contraction that occurs in the dermal layer reduces the open area by pulling adjacent tissue toward the wound center (mediated by fibroblasts’ differentiation into myofibroblasts) [83]. It was demonstrated that AgNPs promote and enhance wound healing through their powerful antibacterial properties, as well as through their ability to decrease inflammation [84,85]. Moreover, it was shown that wound treatment with AgNPs significantly improves the proliferation (differentiation and maturation) of keratinocytes, resulting in healed wounds that closely resemble normal skin, with a relatively thin epidermis and normal hair follicles. On the contrary, AgNPs suppress the proliferation of fibroblasts but promote their differentiation into myofibroblasts, thus accelerating wound contraction and healing. The decrease in collagen production in fibroblasts caused by AgNPs can be useful in anti-fibrosis therapy, leading to wound healing without keloids and scars [83,86]. Galandáková et al. [86] revealed that AgNPs did not show a cytotoxic effect on human dermal fibroblasts and epidermal keratinocytes up to the concentration of 25 ppm, while, for Ag(I) compounds (silver nitrate and silver sulfadiazine), concentrations above 10 ppm are highly toxic and lead to a significant decrease in the number of surviving cells. On the other hand, one investigation showed that the treatment of keratinocytes with 10 ppm of AgNPs reduced the cell viability to 67% after 24 h of exposure, indicating a strong inhibition of HaCaT cell proliferation [87]. Furthermore, human skin is an organ that is constantly exposed to solar ultraviolet (UV) radiation, which can cause skin cell damage. It is well known that, particularly, the UVB component of UV radiation is directly responsible for DNA damage in skin cells, which, if they are not repaired, may ultimately lead to the accumulation of carcinogenic mutations, resulting in malignant transformation [88,89,90]. A research group from the University of Alabama found that AgNPs are efficient as a chemopreventive agent against UVB-induced skin carcinogenesis. Namely, they revealed that the pretreatment of human keratinocytes with AgNPs protects them from UVB-induced DNA damage and significantly reduces the extent of apoptosis of HaCaT cells caused by UVB radiation [89]. Moreover, the same authors investigated the protective effects of AgNPs with different sizes. They observed that the pretreatment of HaCaT cells with AgNPs in the range of 10–40 nm suspends the formation of CPDs (cyclobutane pyrimidine dimers, one of the most lethal DNA lesions induced by UVB radiation), while the AgNPs with diameters of 60 nm and 100 nm did not show any protective effects against UVB-induced DNA damage. The same AgNP size-effect was observed by the monitoring of keratinocyte apoptosis. The pretreatment of HaCaT cells with smaller AgNPs lead to a significant reduction in their apoptosis, compared to nontreated cells, whereas larger AgNPs did not show any protective effect [90].

According to the literature, most of the published results confirm that AgNPs possess very pronounced antimicrobial and anti-inflammatory activities. On the contrary, a significant disagreement is observed regarding the evaluation of the toxic effects of AgNPs on different cell lines. However, all studies agree that the above-mentioned properties are dependent on the shape, size, and concentration of AgNPs, as well as on the exposure time. This study shows that it is possible to achieve and fine-tune the optimal antibacterial activity of Ag-P(NiPAAm/IA) hydrogel nanocomposites, below the cytotoxicity level, without any harmful effects on the surrounding cells.

## 4. Conclusions

In summary, a series of multifunctional Ag-P(NiPAAm/IA) hydrogel nanocomposites were designed for potential skin treatment and produced by a gamma irradiation technique. The microstructural analysis showed a stable porous structure of the hydrogels, which depends on the copolymers ratio. Increases in the IA content significantly increase the porosity and mean pore sizes of P(NiPAAm/IA) hydrogels. Compared with pure polymer matrix, these network parameters decreased upon the incorporation of AgNPs because they act as additional crosslinking points, leading to the formation of a denser polymer network, but without significant influence on the structural parameters. The formation of AgNPs with diameters in the range of 8–16 nm was confirmed. It was revealed that the smallest AgNPs were formed in the less porous P(NiPAAm/IA) (100.0/0.0) homopolymer matrix. The results of swelling measurements indicate that most of the investigated samples uptake fluids from their surroundings by non-Fick diffusion, indicating that the fluid diffusion and polymer chain relaxation are comparable. To evaluate the biomedical potential of Ag-P(NiPAAm/IA) hydrogel nanocomposites, the *in vitro* release of Ag^+^ ions, as well as their antimicrobial activity and cytotoxicity, were investigated. All investigated hydrogel nanocomposites show the initial burst release of a certain amount of silver, followed by slower release. The predominant mechanism of silver release is Fickian diffusion, with a small contribution from chain relaxation. Among the applied models, the Ag^+^ ions’ release is best described by the Peppas–Sahlin model. The antibacterial activity test shows that Ag-P(NiPAAm/IA) hydrogel nanocomposites possess excellent bactericidal effects against both *E. coli* and *S. aureus* bacteria. Moreover, the investigated systems with therapeutically permitted silver concentrations (around 10 ppm) do not exhibit a cytotoxic effect on human HaCaT keratinocyte cells. On the contrary, some extent of HaCaT cell proliferation was observed in these compared to the untreated cells. According to the obtained results, Ag-P(NiPAAm/IA) hydrogel nanocomposites can be considered as efficient therapeutic solutions for the prevention and treatment of bacterial infections and can be applicable in the topical treatment of wound healing.

## Figures and Tables

**Table 1:** Microstructural parameters of investigated hydrogels obtained by micro-CT analysis.

Sample	Porosity (%)	Mean Pore Diameter (μm)	Mean Wall Thickness (μm)	Polymer Volume Fraction (%)	Connectivity Density (1/mm^3^)	Fractal Dimension	Structure Model Index
P(NiPAAm/IA)							
100.0/0.0	80.5 ± 2.8	31.5 ± 1.1	10.1 ± 0.4	19.5 ± 0.7	44,245 ± 1549	2.78 ± 0.13	1.63 ± 0.06
98.5/1.5	84.9 ± 3.2	45.1 ± 1.7	7.9 ± 0.3	15.1 ± 0.6	25,608 ± 973	2.77 ± 0.14	2.13 ± 0.08
97.0/3.0	89.7 ± 4.7	62.0 ± 3.2	7.4 ± 0.4	10.3 ± 0.5	19,512 ± 1014	2.66 ± 0.10	1.63 ± 0.06
95.5/4.5	92.2 ± 4.3	67.3 ± 3.2	6.9 ± 0.3	7.8 ± 0.4	16,847 ± 792	2.69 ± 0.11	1.81 ± 0.07
Ag-P(NiPAAm/IA)							
100.0/0.0	72.5 ± 2.5	19.5 ± 0.7	9.9 ± 0.3	27.5 ± 1.0	69,548 ± 2434	2.81 ± 0.15	1.46 ± 0.05
98.5/1.5	80.0 ± 3.0	29.8 ± 1.1	7.4 ± 0.3	20.0 ± 0.8	66,187 ± 2515	2.78 ± 0.13	1.70 ± 0.06
97.0/3.0	82.4 ± 4.3	36.4 ± 1.9	7.5 ± 0.4	17.6 ± 0.9	45,900 ± 2387	2.77 ± 0.13	1.78 ± 0.07
95.5/4.5	83.2 ± 3.9	41.2 ± 1.9	7.4 ± 0.3	16.8 ± 0.8	45,082 ± 2119	2.78 ± 0.13	1.84 ± 0.08

**Table 2:** Physicochemical parameters of AgNPs in Ag-(PNiPAAm/IA) hydrogel nanocomposites.

Sample Ag-P(NiPAAm/IA)	*r_exp_* (nm) [14]	*N_av_* (at/NPs)	*D_NPs_* × 10^−13^ (NPs/cm^3^)	*C_NPs_* × 10^8^ (mol/cm^3^)	*S.A.* (m^2^/g)	*S_r_* (mg/L)
100.0/0.0	4.08 ± 0.08	16,663 ± 333	10.90 ± 0.57	18.10 ± 0.94	70.0 ± 3.6	0.077 ± 0.005
98.5/1.5	7.22 ± 0.17	92,337 ± 1939	1.84 ± 0.08	3.06 ± 0.13	39.6 ± 1.7	0.028 ± 0.002
97.0/3.0	8.14 ± 0.18	132,324 ± 3308	1.17 ± 0.04	1.94 ± 0.07	35.1 ± 1.2	0.025 ± 0.001
95.5/4.5	7.66 ± 0.17	110,269 ± 2646	1.32 ± 0.05	2.19 ± 0.08	37.3 ± 1.3	0.027 ± 0.002

**Table 3:** Microstructural parameters of the AgNPs in Ag-(PNiPAAm/IA) xerogel nanocomposites.

Sample Ag-P(NiPAAm/IA)	*D_Sch_* (nm)	*a* (nm)	*d* (nm)	*Ɛ* × 10^4^	*σ* (GPa)	*δ_D_ ×* 10^−15^ (1/m^2^)
100.0/0.0	8.47	0.4090	0.2362	6.78	0.056	5.25
98.5/1.5	14.83	0.4098	0.2366	−3.85	−0.032	4.49
97.0/3.0	15.63	0.4102	0.2368	−3.69	−0.031	3.97
95.5/4.5	15.61	0.4105	0.2370	−3.28	−0.027	3.63

**Table 4:** Parameters of swelling process of P(NiPAAm/IA) hydrogels and Ag-P(NiPAAm/IA) hydrogel nanocomposites in distilled water and SBF at 37 °C.

Sample	Swelling Medium					
	H_2_O			SBF		
	*SD_eq_*	*n*	*D* (cm^2^/s)	*SD_eq_*	*n*	*D* (cm^2^/s)
P(NiPAAm/IA)						
100.0/0.0	0.3	0.36	4.54 × 10^−7^	0.2	0.52	5.53 × 10^−6^
98.5/1.5	4.2	0.47	1.63 × 10^−6^	2.5	0.59	1.48 × 10^−5^
97.0/3.0	73.7	0.87	4.00 × 10^−5^	7.0	0.61	2.41 × 10^−5^
95.5/4.5	113.3	0.88	3.64 × 10^−5^	12.0	0.84	5.65 × 10^−5^
Ag-P(NiPAAm/IA)						
100.0/0.0	0.3	0.35	3.83 × 10^−7^	0.2	0.51	7.90 × 10^−6^
98.5/1.5	0.4	0.36	3.07 × 10^−7^	1.7	0.56	2.06 × 10^−6^
97.0/3.0	0.5	0.53	1.38 × 10^−6^	5.7	0.63	1.29 × 10^−5^
95.5/4.5	0.7	0.68	3.90 × 10^−5^	10.1	0.64	1.61 × 10^−5^

**Table 5:** Fitting parameters of cumulative silver release from Ag-P(NiPAAm/IA) hydrogel nanocomposites obtained by first-order, Higuchi, Korsmeyer–Peppas, Kopcha, and Peppas–Sahlin models.

Sample Ag-P(NiPAAm/IA)	First-Order		Higuchi		Korsmeyer-Peppas		
	*k*_1_ × 10^6^ (1/s)	*R* ^2^	*k_H_* × 10^2^ (1/s^1/2^)	*R* ^2^	*k_KP_* × 10^3^ (1/s^n^)	*n*	*R* ^2^
100.0/0.0	2.15	0.833	2.52	0.976	0.96	0.59	0.990
98.5/1.5	3.24	0.917	2.99	0.979	0.83	0.63	0.962
97.0/3.0	2.90	0.908	2.90	0.977	1.10	0.61	0.952
95.5/4.5	3.68	0.949	3.11	0.975	1.42	0.58	0.954
	**Kopcha**			**Peppas-Sahlin**			
	***A* × 10^3^** **(1/s^1/2^)**	***B* × 10^6^** **(1/s)**	***R* ^2^**	***K*_1_ × 10^2^** **(1/s^m^)**	***K*_2_ × 10^5^** **(1/s^2m^)**	***m***	***R* ^2^**
100.0/0.0	2.11	−1.90	0.976	0.65	1.71	0.40	0.990
98.5/1.5	2.37	−1.97	0.967	1.06	3.16	0.36	0.989
97.0/3.0	2.34	−2.00	0.930	1.87	7.42	0.30	0.976
95.5/4.5	2.37	−1.85	0.930	2.43	6.99	0.26	0.977

## Data Availability

The original contributions presented in the study are included in the article, further inquiries can be directed to the corresponding author.
